# Diagnosing Posttraumatic stress disorder (PTSD) in people with dementia

**DOI:** 10.1192/j.eurpsy.2023.1996

**Published:** 2023-07-19

**Authors:** S. Sobczak

**Affiliations:** Neuropsychology and Psychopharmacology, Faculty of Psychology and Neuroscience, Maastricht University, Maastricht, Netherlands

## Abstract

**Introduction:**

Traumatic stress is a major determinant of decreasing global mental health (Schnyder, 2013). Traumatic stress is a global public health issue, with the majority (70.4% and 88.7%) of the general population experiencing a traumatic event at least once in their lifetime (Schnyder, 2013, Kessler et al., 2017, de Vries and Olff, 2009, Ribeiro et al., 2013), which may result in Posttraumatic Stress Disorder (PTSD) (Iqbal et al., 2022, Riedl et al., 2019). People with a diagnosis of PTSD have been found to be at increased risk of developing dementia (hazard ratio (HR) of 1.61)(Gunak et al., 2021). Studies on the comorbidity of PTSD in dementia are sparse, probably, because of the lack of a valid diagnostic tool (Havermans et al., 2022). As subjects with dementia are often unable to give a valid report of their life history, in particular a delayed-onset PTSD may be easily missed. Preliminary findings reported a comorbidity rate of PTSD in veterans with dementia between 4.7- 7.8% (Sobczak et al., 2021).

**Objectives:**

We will investigate a) clinical manifestation and b) diagnostic challenges of PTSD in dementia.

**Methods:**

a) A structured review with a PRISMA design, b) a qualitative case-study will be used.

**Results:**

a) 13 papers were included. Only 1% of included cases fulfilled the DSM-5 criteria of PTSD.

Most commonly described PTSD symptoms were: irritability and anger (9%), persistent negative emotional state ( 9%), and sleep disturbances (8%). In 93% of the cases reports, other symptoms were described, e.g. screaming (33.3%). B) Diagnostic challenges are: attributing symptoms to the past traumatic event, overlap in symptoms between PTSD and personality disorders and interference of other neuropsychiatric symptoms.

**Conclusions:**

Mondial the number of people with dementia are sky rocketing and number of subjects with comorbid PTSD is substantial and will increase steadily in the coming years. PTSD and dementia are both known for their impact on the quality of life of those affected. In clinical practice, we see that the combination of both causes significant psychological suffering. And that while it is precisely in people with dementia that the experiences of these violent events often revive.

As the clinical manifestation of PTSD in dementia may differ and expertise on PTSD is often missing in geriatric wards, diagnosis of PTSD is easily missed. Hence, related behavior is often described as ‘unexplained’ or ‘problem’ while it can better be seen as ‘signal’ behavior (van Dongen et al., 2022). Adding observational research to the diagnostic process in people with dementia can give insight into underlying causes of neuropsychiatric symptoms. The TRADE-interview can be helpful in diagnosing PTSD. The value of using validated tests is emphasized.

**Disclosure of Interest:**

None Declared

